# Manuka honey microneedles for enhanced wound healing and the prevention and/or treatment of Methicillin-resistant *Staphylococcus aureus* (MRSA) surgical site infection

**DOI:** 10.1038/s41598-020-70186-9

**Published:** 2020-08-06

**Authors:** Galit H. Frydman, David Olaleye, Damodaran Annamalai, Kim Layne, Illina Yang, Haytham M. A. Kaafarani, James G. Fox

**Affiliations:** 1 Division of Comparative Medicine and Division of Biomedical Engineering, Department of Biological Engineering, Massachusetts Institute of Technology, 500 Technology Square, 3rd Floor Rm 383, Cambridge, MA 02139 USA; 2 BioMEMs Resource Center, Massachusetts General Hospital, Charlestown, MA USA; 3 Division of Trauma, Emergency Surgery & Surgical Critical Care and Department of Surgery, Massachusetts General Hospital, Boston, MA USA

**Keywords:** Drug delivery, Biomedical engineering

## Abstract

Manuka honey (MH) is currently used as a wound treatment and suggested to be effective in Methicillin-resistant *Staphylococcus aureus* (MRSA) elimination. We sought to optimize the synthesis of MH microneedles (MHMs) while maintaining the MH therapeutic effects. MHMs were synthesized using multiple methods and evaluated with in vitro assays. MHMs demonstrated excellent bactericidal activity against MRSA at concentrations ≥ 10% of honey, with vacuum-prepared honey appearing to be the most bactericidal, killing bacterial concentrations as high as 8 × 10^7^ CFU/mL. The wound-healing assay demonstrated that, at concentrations of 0.1%, while the cooked honey had incomplete wound closure, the vacuum-treated honey trended towards faster wound closure. In this study, we demonstrate that the method of MHM synthesis is crucial to maintaining MH properties. We optimized the synthesis of MHMs and demonstrated their potential utility in the treatment of MRSA infections as well as in wound healing. This is the first report of using MH as a substrate for the formation of dissolvable microneedles. This data supports the need for further exploration of this new approach in a wound-healing model and opens the door for the future use of MH as a component of microneedle scaffolds.

Surgical site infections (SSIs) have historically been associated with increased morbidity and mortality but remain an issue in modern day healthcare. As of 2017, the Centers for Disease Control and Prevention (CDC) estimated that SSIs occurred in at least 1.9% of all surgical patients; however, this number is most likely not representative of the total number of SSI cases since about 50% of SSIs occur after hospital discharge^1,2^. MRSA is the most common cause of SSIs leading in an increased morbidity in patients and increased hospital charges^3,4^. In a study evaluating the 90-day mortality rate in patients with SSI, it was discovered that patients with a methicillin-resistant *Staphylococcus aureus* (MRSA) SSI had a 3.40 times increased mortality rate (95% CI, 1.5–7.2) than patients infected with a methicillin-susceptible *S. aureus* strain^5^. Given the health and economic burden attributable, in particular, to SSIs caused by antimicrobial-resistant organisms such as MRSA, there is a critical need for the development of novel treatment options that can address resistant organisms.

Manuka honey comes from New Zealand and Australia. It is harvested by European honeybees (*Apis mellifera)* that have pollinated and collected nectar primarily from the Manuka tree (*Leptospermum scoparium*). Honeys have natural antimicrobial, immuno-stimulant and wound healing properties due to a number of factors, including: providing a moist environment, high sugar concentration, low pH, antimyeloperoxidase activity, polyphenolic compounds, Bee defensing-1, hydrogen peroxide, enzymes, including invertase, amylase, glucose oxidase, catalase and methylglyoxal (MGO)^6–11^. MGO is one of the major antibacterial components in honey and, while MGO is present in most honeys in small quantities, it is present at the highest concentrations in Manuka honey, as it is derived from dihydroxyacetone which is found in high concentrations in the nectar of Manuka flowers. One of the reasons that Manuka honey is thought to be such a potent antimicrobial agent as compared to other honeys is because the main type of antibacterial activity in other honeys is peroxidase-based, which is thought to be neutralized by catalases present in blood, serum and wound tissues, whereas MGO remains active^10–12^. Unique Manuka Factor (UMF) is the standardized terminology used to represent the concentration of MGO in the honey, with a minimum grade of 10 UMF typically considered to be “Active Manuka Honey”^13,14^. MGO has been shown to have non-peroxidase antibacterial activity with effectiveness against some biofilms^15^. It is also noteworthy that there is currently no evidence of antibiotic resistance to Manuka honey^9^. Manuka honey has been used in medicine for centuries and the first honey-based product for wound dressings was approved by the FDA in 2007 (MEDIHONEY, DermaSciences, Plainsboro, NJ, USA)^16^.

Microneedles are a growing tool in the medical community. They are minimally invasive devices that are able to penetrate the skin and assist in drug delivery^17,18^. In dermatology they’ve also been shown to promote healing of scar tissue^19,20^. They are intended to only penetrate to about 50–100 µm into the skin, and not stimulate the nerves, enabling them to be painless. While microneedles are more commonly made out silicon, metals, ceramics, or silica glass, those made of carbohydrates have several advantages^21^. The drugs can be incorporated into the mixture before molding the microneedles, and the microneedles are able to dissolve into the skin after application. To our knowledge, although sugars are a common scaffold for microneedle synthesis, honey has not yet been explored as a microneedle building-block or significant component.

For this project, Manuka honey is used for the synthesis of Manuka honey microneedles (MHMs). In this set of experiments, we optimize a method by which to synthesize MHMs, and used a series of in vitro experiments to test and optimize the properties of the MHMs in order to maintain the Manuka honey’s wound-healing and bactericidal properties.

## Methods

### Manuka honey microneedle synthesis

**Figure 1:**
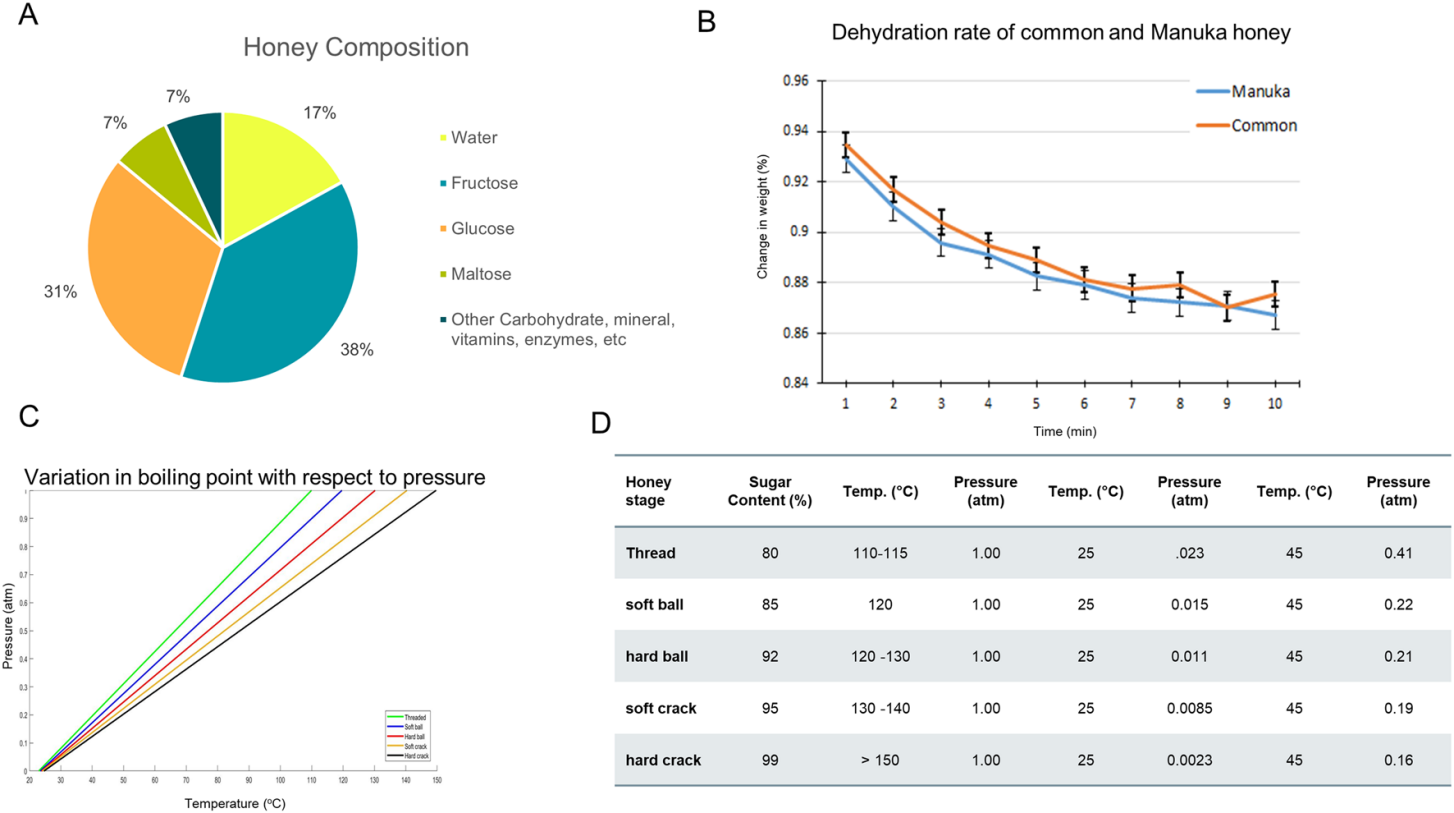
Characterization of Manuka honey. (**A**) A pie chart demonstrating the previously-reported general composition of honey^28,29^. (**B**) This graph represents the dehydration kinetics of common table honey and Manuka honey over 10 min at 80 °C. (**C**) This simulation demonstrates the relationship between temperature and pressure to achieve the various stages of honey dehydration. (**D**) Table containing selected temperature and pressure conditions, demonstrating the relationship and conditions required to achieve the various dehydration states of honey.

Honey typically has a water concentration of about 17% (Fig. 1A) and goes through a number of phase transitions that are typically described in the making of candy (Fig. 1D). Because Manuka honey has various active enzymes and chemicals that we would like to preserve in the Manuka honey microneedles (MHMs), and high heat can have deleterious effects on these, we needed to characterize the Manuka honey and derive the pressure that was required to sufficiently dehydrate the honey to form a “hard crack” (HC) stage candy within a microneedle mold. For these experiments, we utilized Manuka Honey Sterile gel (Medihoney, Derma Sciences, Plainsboro, NJ) and common table honey (clover-based). Medihoney has been reported to have an MGO concentration of about > 514 mg/kg (UMF > 15), while it is estimated that the common table honey has low to no detestable MGO (UMF < 5)^14,15,22,23^. The Medihoney is also gamma irradiated prior to packaging in order to eliminate any pathogens, such as *Clostridium botulinum* spores.

In order to characterize the percent water composition of the honey, equal volumes of both common table honey and Manuka honey were exposed to 80 °C and weighed every minute until they reached the HC phase (i.e. the phase at which we assume all the moisture content is gone). Achievement of the HC phase was determined by placing the honey in cool water and then applying pressure to the honey, demonstrating hardness, lack of deformability, and a “crack” when enough pressure was applied. This information was then used to calculate the pressure that the honey would need to be exposed to at various temperatures in order to achieve HC.

Through the use of the Clausius–clapeyron equation, we can estimate that the approximate pressure that would be necessary to boil water at 40 °C would be around 10% of atmospheric pressure.

Clausius-clapeyron Eqn:

1 $$ln\left(\frac{{P}_{2}}{{P}_{1}}\right) = \frac{-\Delta {H}_{vapor}}{R}\left(\frac{1}{{T}_{2}}-\frac{1}{{T}_{1}}\right)$$

Solving for P_2_ which represents the final pressure.

2 $${P}_{2}= {P}_{1}{e}^{ \frac{-\Delta {H}_{vapor}}{R}\left(\frac{1}{{T}_{2}}-\frac{1}{{T}_{1}}\right)}$$

The moles of water and sugar are then used to calculate the molar fraction of water in the honey.

3 $${X}_{{H}_{2}O}= \frac{moles {H}_{2}O}{moles sugar + moles {H}_{2}O}$$

This molar fraction of water is then applied to Raoult’s law in order to calculate the pressure required to reach the boiling point of the honey.

4 $${P}_{Honey,Boiling}={X}_{{H}_{2}O} \times {P}_{{H}_{2}O, Boiling}^{O}$$

From this equation we can deduce that, as the concentration of water in honey decreases through boiling, the pressure that the honey is under will also have to decrease to be low enough for it to continue boiling. This gave values that are meant to outline what pressures the water will begin to boil at for our dehydration; this isn’t necessarily the limit of what we need, but the starting pressure. We use this approach to estimate the temperature and pressure combinations that would be required to produce HC MHMs. For the initial pressure and temperature variation experiments, we used a TECA hotplate and a desiccator with a RS1.5 4CFM vacuum pump (Emerald Gold). Once the protocol was optimized, we used a NAPCO Vacuum oven (Model 5831) with manually-set temperature and pressure settings.

**Figure 2:**
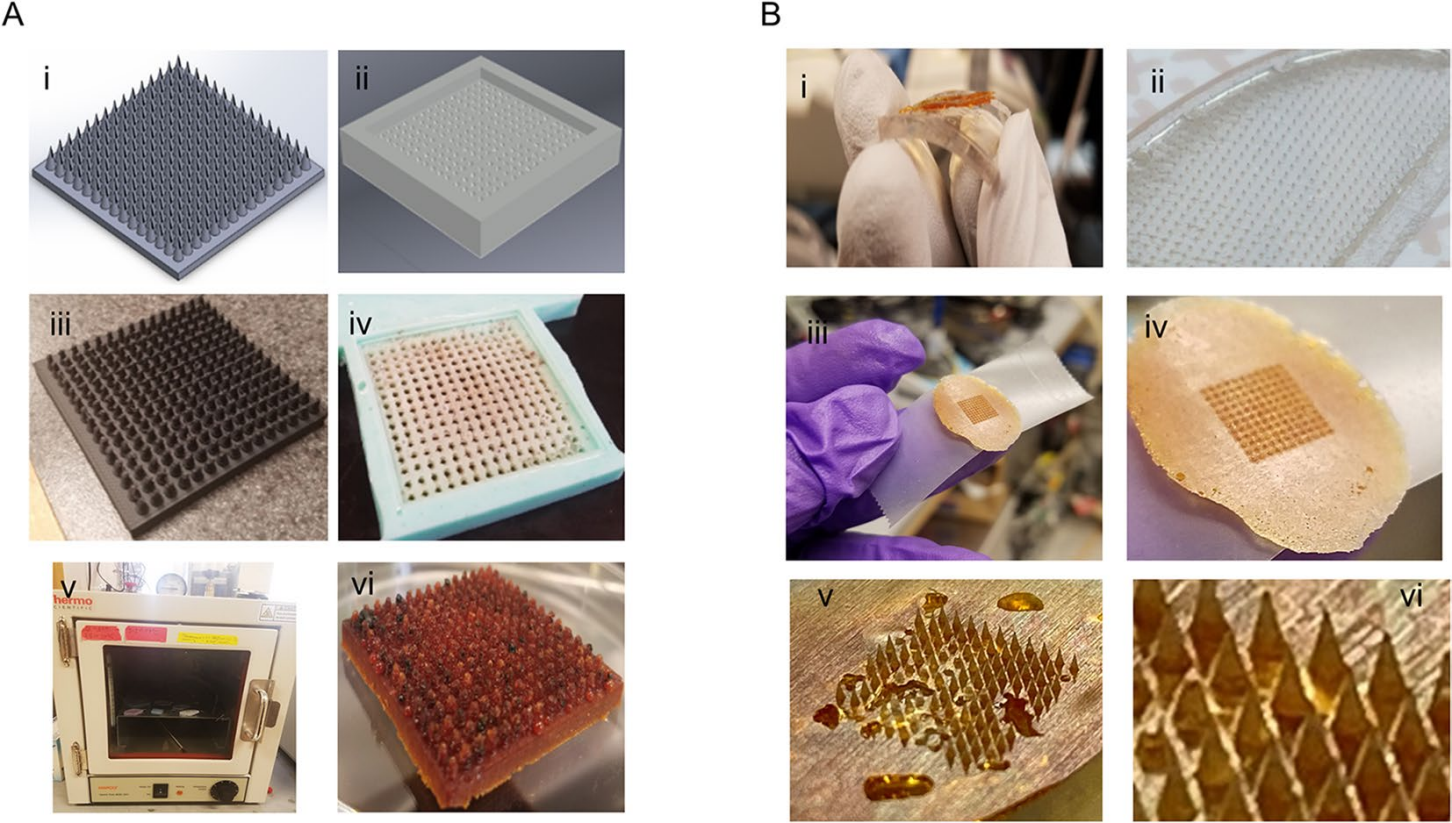
Manuka honey microneedle (MHM) synthesis. (**A**) Autocad rendering of the microneedle molds (i–ii) with their respective 3D-printed (iii) and elastomer negative mold (iv). The vacuum oven (v) used to make the honey microneedles (vi) is also shown. These molds, although functional, resulted in uneven dehydration rates and assymetrical microneedles. (**B**) PDMS was later used as a mold, which allowed for fine feature formation and ease of honey removal (i–ii). Microneedle molds were made from two commercially-available microneedle patches using PDMS, and both resulted in fine and even microneedles (iii–vi).

We then used the resulting information to create MHMs using both, custom and commercially-available microneedle molds. In order to make the in-house microneedle molds, AutoCad (Autodesk, San Rafael, CA) was used to create a microneedle match 3D model and the negative mold was derived from the positive mold (Fig. 2A). The positive mold was printed in acrylonitrile butadiene styrene (ABS), using a Stratasys uPrint 3D printer (Prairie, MN). This was then used to make a negative mold out of silicon. This negative mold was the one used in experiments for the testing and optimization of the temperature and pressure combinations for transforming the honey into the HC phase within a mold. Once this was optimized, we tested this protocol using smaller molds made of polydimethylsiloxane-diacrylamide (PDMS, Sylgard™ Silicone Elastomer Kit, DOW Corning, Midland, MI) (Fig. 2B). The PDMS allowed for the creation of smaller features and easy removal of the MHMs from the negative mold. For the PDMS molds, we created one in-house negative mold using a commercially-available microneedle patch as the positive mold (Hyaluronic Acid Micro Needle Patch, WELLAGE) and we also purchased a microneedle negative PDMS mold, 11 × 11 array with 600 um height, 300 um base diameter, and 5 um tip radius from Blueacre Technology (Co Louth, Ireland). The final conditions for the cooked- and vacuum-prepared conditions were 165 °C at 1 atm, and 40 °C at 0.16 atm for 20 h, respectively.

### Bacterial killing assay

**Figure 3:**
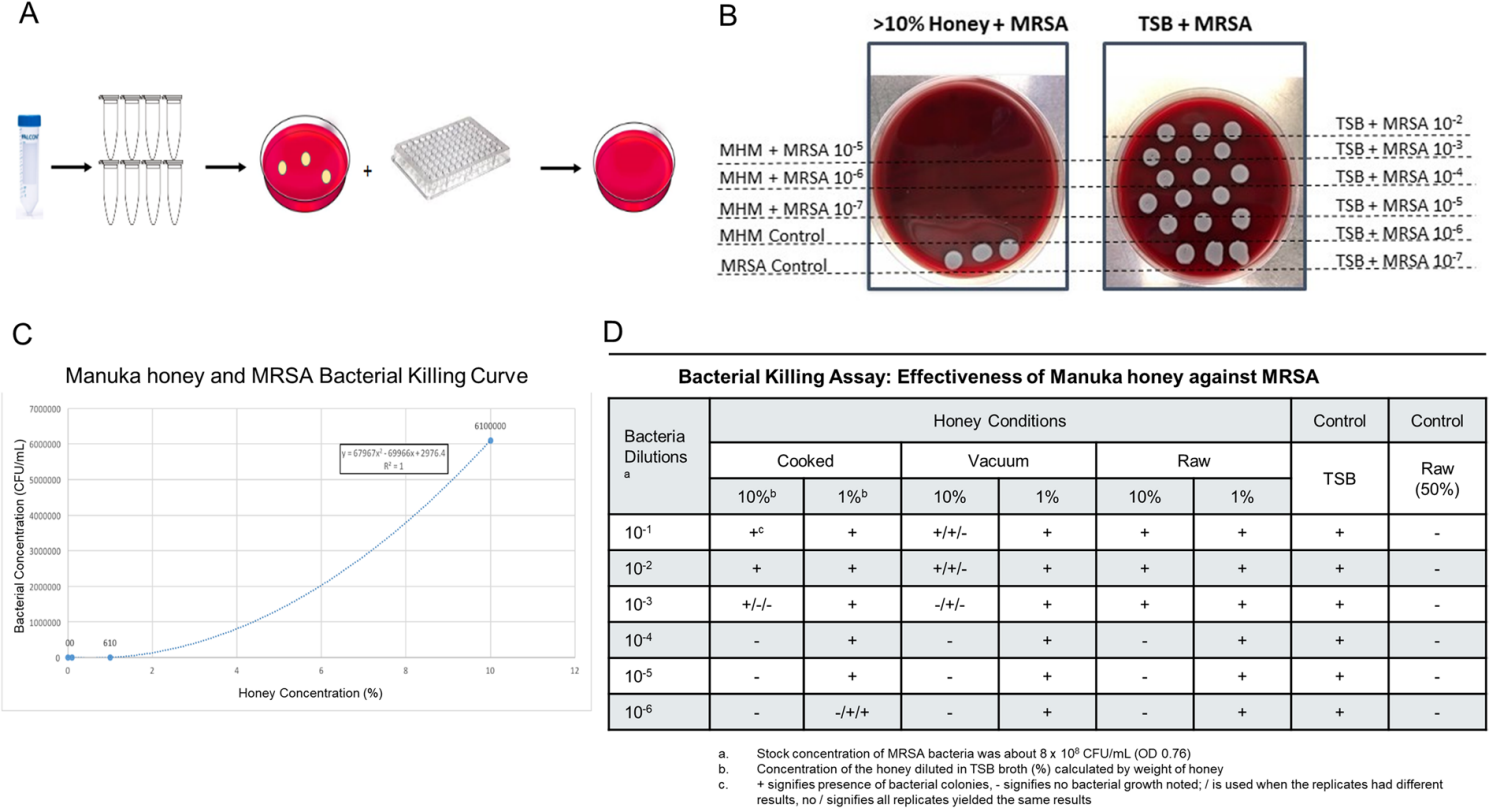
Antibacterial effects of Manuka honey. (**A**) Flow diagram of the experimental set-up for the co-culture of MRSA bacteria and Manuka honey preparations. First the bacteria and honey conditions are mixed and serial dilutions are made. One aliquot of these dilutions are plated to verify growth and purity of the bacterial culture and the other aliquot is seeded in a 96-well plate for overnight culture. The following day, these are plated and cultured overnight. (**B**) Representative photographs of a > 10% Manuka honey positive control on the left and a negative control, TSB only, on the right. As expected, there is no growth in the honey conditions but there is copious growth in the negative control. (**C**) A bacterial killing curve was graphed based on the 24-h colony counts of various concentrations of Manuka honey and MRSA. (**D**) Table of the colony counts for each condition in triplicate demonstrating bactericidal properties of Manuka honey at 10% but not at 1%. "+" represents bacterial growth and "−" represents no bacterial growth. Single “ + ” and “–” represents consistent results between replicates, while “ + ” and “–” separated by “/” indicates variable results between replicates.

For the bacterial killing assay, 10% and 1% of honey solutions were incubated with methicillin-resistant *S. aureus* (MRSA) (supplied by the Division of Comparative Medicine, MIT) to assess for bacterial killing efficacy (Fig. 3A). Briefly, MRSA was cultured in Trypsin Soy Broth (TSB) overnight to reach optimal growth phase. The MRSA broth was diluted with TSB until an O.D. of 0.76 in order to make the stock solution of the bacteria (~ 8 × 10^8^ CFU/mL). Serial dilutions of the bacterial stock were made in various culture broth conditions: TSB (negative control), 1% vacuum, cooked, and raw honey, 10% vacuum, cooked, and raw honey, and 50% raw honey (positive control). Earlier experiments demonstrated that honey did not have a bactericidal effect when immediately plated and cultured; indicating that honey requires some co-incubation with the bacteria and kills in a time and concentration-dependent manner. For this set of experiments, 80 µL of each condition was plated in a 96-well plate and incubated overnight at 37 °C and one aliquot is directly plated on blood agar and incubated overnight to verify culture purity. The following day, each condition was plated onto a blood agar culture dish, using 10 µL spots in triplicate, incubated at 37 °C for 24 h and then plates were imaged and assessed for bacterial growth (Fig. 3B). These experiments were run in triplicate. One of the raw honey conditions had a fungal contaminant, therefore only duplicate of this condition was evaluated. The goal of this experimental design was to explore the maximum bacterial burden (concentration) that the specific honey preparation could maintain adequate antibacterial properties against. The purpose of this is to mimic various wound environments that may have bacterial concentrations ranging from “contaminated” (< 10^4^ CFU/mL) to “infected” (> 10^5^ CFU/mL)^24–26^. Although various concentrations of bacteria were explored, only 10% and 1% honey concentrations were evaluated in this set of experiments, limiting the ability to experimentally determine the minimum inhibitory concentration (MIC) of the honey preparations.

### Wound healing assay

**Figure 4:**
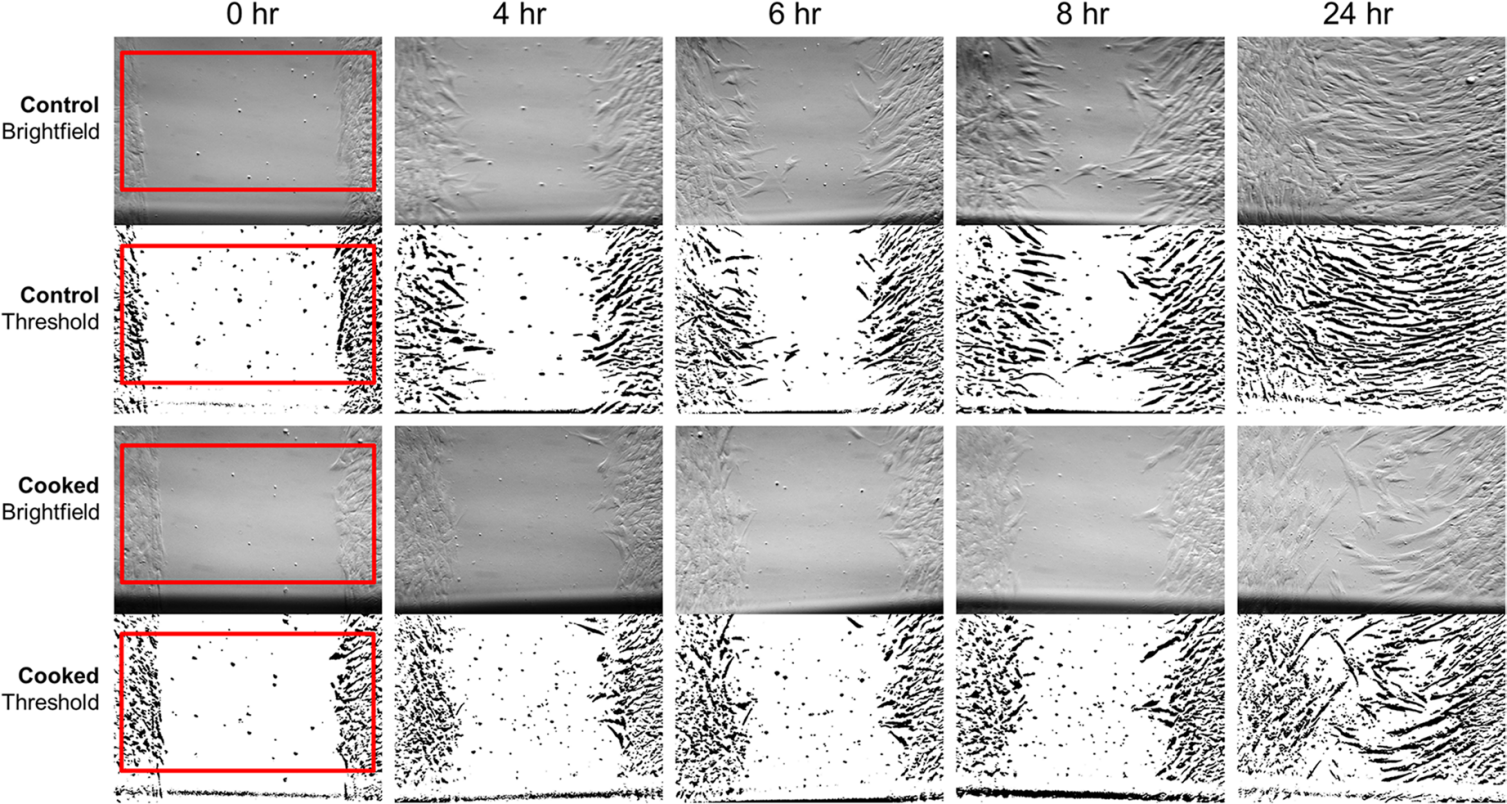
Wound-healing assay image analysis. Representative bright field microscopy images (10x) of a single set of insert wound healing experiments. The top two rows are the negative control condition and the bottom two rows are the cooked condition. The second and fourth row are the corresponding thresholded images that were used for the image analysis, where the percent wound closure was calculated based on the change in black-to-white ratio in the image. Bottom black line in each image is the black marker used for orientation and image analysis. The red squares on the left are representative areas that were included in the image analysis. The black marker line area was not included so as to not skew the results.

For the wound healing assay, human dermal fibroblasts were cultured with 0.1% of raw, cooked, and vacuum-prepared honey to assess their effect on wound closure. Two wound closure models were used: scratch and insert model. Briefly, normal human dermal fibroblasts (ATCC, PCS-201-012), were cultured overnight in fibroblast basal medium (ATCC, PCS-201-030) with low serum (ATCC, PCS-201-041). The media was removed, and trypsin was used to detach the cells from the culture flask. The cells were then centrifuged at 1,200 rpm for 2 min and the supernatant was discarded. A cell suspension of 7 × 10^5^ cells/mL was prepared. A black line was drawn on the bottom of a 12-well plate to allow for location identification during subsequent image analysis. For the insert assay, a 2-well insert (ibidi, Madison, WI) was placed on the bottom of the well. 70 µL of the suspension was added to each well, along with the insert. The cells were incubated for 36 to 48 h to become confluent and then the 2-well insert was removed from the insert well. For the scratch assay, 2 × 10^5^ cells/ml was prepared and 1 mL of cells per well was plated in 24 well plates and incubated for 36 to 48 h to become confluent. Then, a 200 μl pipette tip was used to create scratch. For both the insert and scratch assay, the media is then removed from each well and washed twice with growth media to remove the detached cells. Then 1 mL of the growth media with 0.1% of raw, cooked, and vacuum-prepared honey was added. Pictures were taken at time 0, 4, 8, and 24 h (6 h for the insert condition). Each experiment was done in triplicate. For the image analysis, Image J was used to create thresholded, black and white images. These were then used to calculate the percent of white and black space (representing the cell migration) in the images (Fig. 4).

### Statistical analysis

Statistical analysis and graphs of the various experiments presented in this study was performed using Microsoft Excel. Descriptive statistics performed included calculating of the means, standard deviation, standard error, percent change from baseline. These various parameters were then analyzed with a paired t-test with a p-value of 0.05 being considered significant.

## Results

### Manuka honey microneedle synthesis

While boiling (cooking) the honey will decrease the water content sufficiently to reduce it to a HC state, we also explored the use of other synthesis methods, such as dehydration (vacuum), in order to avoid the degradation or inactivation of bioactive molecules within the honey that may be heat-sensitive. Manuka honey is a thixotropic substance^27^. This means that it is a non-Newtonian fluid that has a lower viscosity when disturbed, undergoing shearing stresses. Honey is typically transitioned from a liquid form into a solid form through the application of high heat. While there are many ways to transform honey into hard microneedles, we tested two synthesis methods: cooking and vacuuming. While evaluating these methods, we make the assumption that the thixotropic properties of the honey aren’t significant when in low enough quantities and its behavior can still be approximated by more Newtonian mechanics. First, the boiling point of water under different pressures is calculated in order to approximate the changes an increased sugar concentration would result in. Next, the concentration of water in honey, both table and Manuka, was calculated to be approximately 17% and both of the honey’s appeared to dehydrate at the same rate (Fig. 1B). This experiment was necessary for being able to approximate the impact of increased sugar concentrations on the boiling point of the honey. By assuming that the behavior of the honey is similar enough to a Newtonian fluid when in small enough quantities, Raoult’s law (Eq. 4) can be applied to make an approximation for how much pressure the honey can have and dehydrate to various states (Fig. 1C,D).

For example, in order to calculate the mole fraction of water in honey, we use total moles of sugar, based on the previously-reported basic composition of honey (Fig. 1A), 23.957 mol sugar. And the total moles of water in the honey is 55.56 mol water^28,29^. Therefore:

5 $${X}_{{H}_{2}O}= \frac{55.56}{23.957 + 55.56}$$

This molar fraction of water is then applied to Raoult’s law in order to calculate the pressure required to reach the boiling point of the honey.

6 $${P}_{Honey,Boiling}=0.7 \times 22 torr$$

Therefore, the pressure at which honey boils at room temperature (25 °C) is 15.4 torr (0.023 atm). After assessing the various temperature and pressure combinations experimentally, we used a temperature of 40 °C and a pressure of 121.6 torr (0.16 atm) over 20 h to produce the vacuum-prepared MHMs. The cooked MHMs were prepared using a temperature of 165 °C and a pressure of 760 torr (1.00 atm). We were able to successfully synthesize MHMs from various negative molds using these conditions (Fig. 2).

### Bacterial killing assay

The effect of various conditions of MHMs were assessed in a bacterial killing assay. After 24 h of MRSA and MHM co-incubation, it was found that the various preparations of the MHMs had an effect on the bacterial-killing capabilities of the honey. A raw honey bacterial killing curve was first evaluated (Fig. 3C), where multiple concentrations of raw Manuka honey were co-incubated with MRSA bacteria and then colonies were counted after a second 24-h incubation. Based on best-fit line equation, we expect for 10% and 1% raw Manuka honey to start having bacterial-killing abilities at about 6 × 10^6^ CFU/mL and 6.1 × 10^2^ CFU/mL, respectively. The positive control for the experiments was 50% raw Manuka honey, which killed all bacteria after a 24-h incubation period. The experimental results of MRSA assay shows that all 10% honey preparations start killing bacteria at about 8 × 10^4^ CFU/mL, with the vacuumed honey having variable killing results up to 8 × 10^7^ CFU/mL, as depicted by inconsistent killing results in the replicates as indicated by the “ + ” (growth) and “−” (no growth) symbols (Fig. 3D). These results suggest that at least a 10% honey concentration is needed for bactericidal effects on MRSA, there is an initial bacterial-concentration-relationship, and the vacuumed honey may have more potent bactericidal effects than the raw and cooked honey preparations. These results also suggest that local administration of a minimum of 10% concentration MHM would have sufficient antibacterial properties for an “infected” wound, which is estimated to have a bacterial concentration of ≥ 1 × 10^4^ CFU/mL (most commonly > 1 × 10^5^–1 × 10^6^ CFU/mL). ^24^.

### Wound healing assay

**Figure 5:**
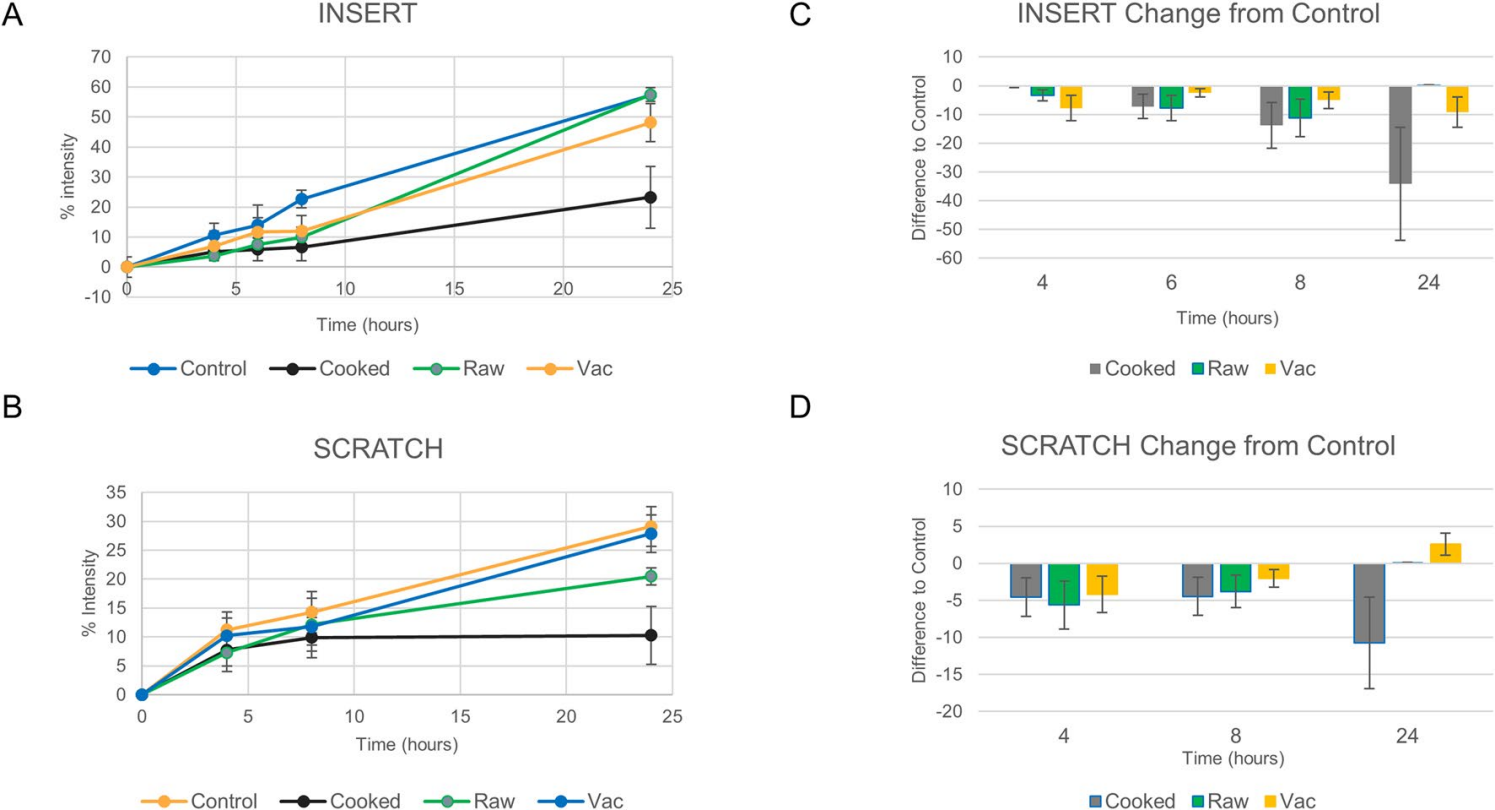
Effect of Manuka honey on wound healing. Analysis of the insert and scratch wound-healing assays are shown here. A line graph showing the mean with the standard deviation for each condition demonstrates that the cooked condition has significantly less closure than the raw and vacuumed conditions (**A**, **B**). Bar graphs of the percent change in wound closure, normalized to time 0 h, confirms this significant difference between the cooked honey and the raw and vacuumed honey (**C**, **D**). In the scratch assay, the vacuumed honey appears to trend towards even faster healing than the control or the other honey conditions.

**Figure 6:**
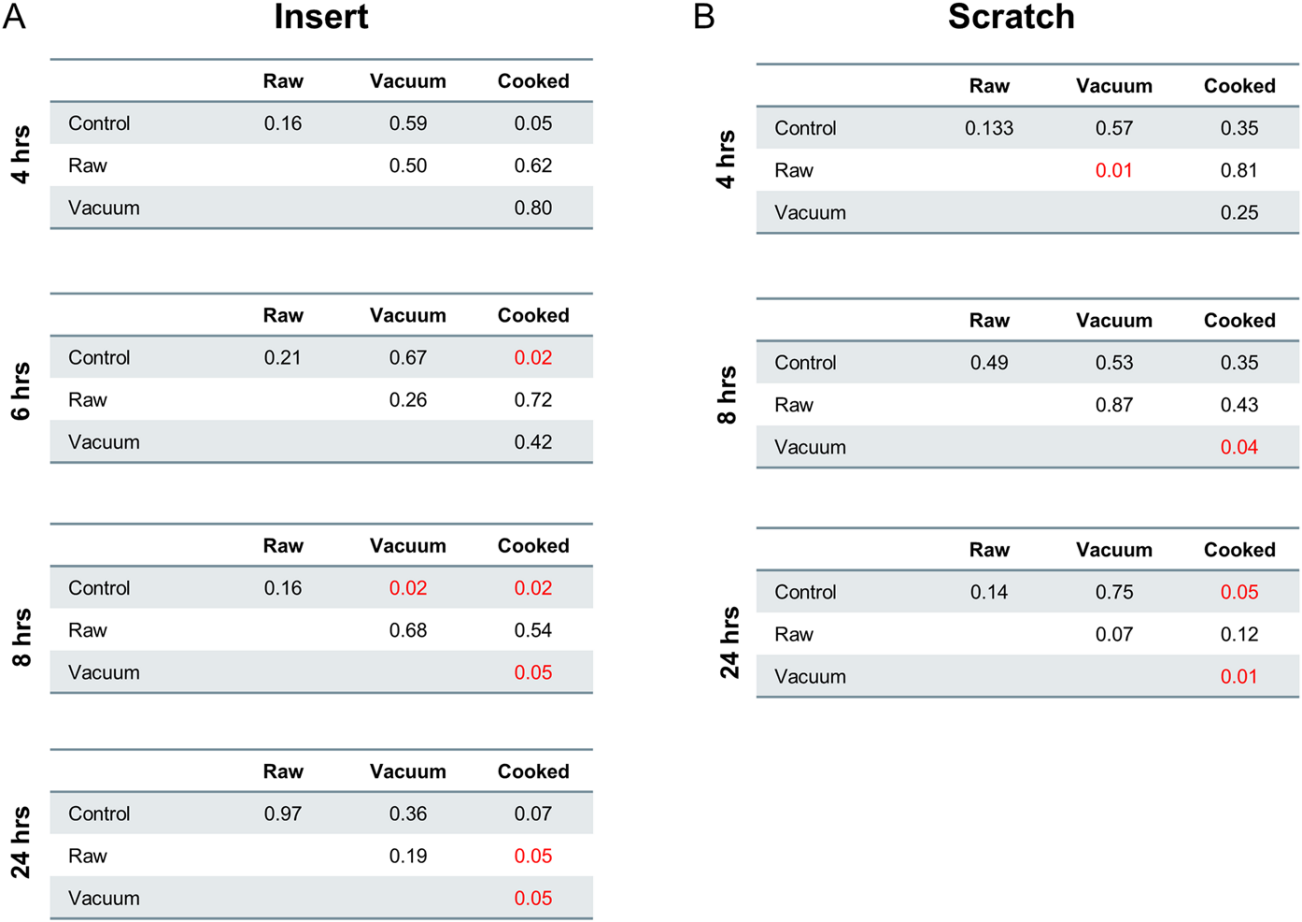
Wound healing statistical analysis. Paired *t* tests for each honey condition at each time point for both the insert (**A**) and scratch (**B**) wound-healing assays was calculated. Values in red have a *p* value ≤ 0.05 and are considered statistically significant.

The effects of various preparations of MHMs on two wound healing assays, scratch and insert assays, was assessed. Earlier experiments confirm that high concentrations of honey, > 1%, appears to be cytotoxic, while concentrations ≤ 0.1% appear to be safe. This is consistent with previous literature; therefore, 0.1% honey concentration was used for this set of experiments^30^. Two assays were performed in order to assess the differences, if any, in the effects of the honey preparation on in vitro wound healing models with different cellular behaviors. In the scratch assay, cell-injury markers are released as a results of the physical scraping and cell damage of the pipette tip; while in the insert assay, a simple cell migration model without the cell injury markers is evaluated. Both the insert and scratch assay model demonstrated significantly reduced wound-gap closure in the cooked honey condition based on analysis of the gap-closure status at the 24-h end point (Figs. 5 & 6). In the scratch assay, the vacuum-prepared honey appears to show a trend towards faster wound gap closure than even the raw honey condition. These results demonstrate that the preparation of honey does have an effect on the wound-healing properties of the honey and that the cooked honey can denature the beneficial proteins and materials in the honey and be detrimental to wound healing. We also observed that there appeared to be different cell migration trends, such as a decrease in cell migration at time point 8 h in the raw and vacuumed conditions of the insert assay, despite potentially reaching the same final 24-h endpoint closure (Fig. 5A). This may indicate differences in cell activation and signaling pathways in the honey-treated cells as compared to the control cells.

### Discussion

In this paper, we present a set of in vitro studies optimizing the synthesis of microneedles made from Manuka honey (MHMs) while maintaining their antibacterial and wound-healing properties. Manuka honey has traditionally been used as a holistic treatment for everything from topical wounds, sore throats, and as an adjunct to cancer treatment^8,11,31,32^. Although once considered “alternative”, the many unique medicinal properties of Manuka honey have captured the attention of modern medicine^8,33^. The particular use of honey in the treatment of infected and non-healing wounds is particularly interesting; especially when honey resistance training studies demonstrate that, at certain concentrations, bacteria do not develop resistance and are incapable of proliferating^9^.

Manuka honey is a thixotropic material and is amongst the highest viscosity amongst a range of honeys^27,34–37^. The process of dehydrating honey is usually done with high heat, which likely results in damage to the honey’s valuable chemicals and enzymes^38,39^. Dehydration of the honey to a supersaturated sugar solution at hard crack (HC) phase (≤ 1% moisture content) is essential in the synthesis of microneedle patches, which will require the tensile strength required to penetrate skin (or other tissues); the HC state’s key characteristics are brittleness and rigidity. At this stage the honey’s stickiness is at a minimum and it’s easier to apply or use as a solid object for tissue penetration. The task of dehydrating honey is analogous to candy making. In order to reduce the amount of heat required to dehydrate the honey, we calculated and optimized a protocol for moisture content reduction that utilizes low temperature and low pressure (vacuum). Although the vacuum method was successful in bringing the honey to HC, a series of experiments were performed to verify that the most desired properties of the honey were not affected.

Due to the complex, and valuable, chemical and enzymatic composition of honey, we evaluated various preparation methods for the synthesis of MHMs and tested their respective in vitro effects on MRSA bacteria and wound-healing models. In this study, we identified that the preparation of the honey has a significant effect on the biologic properties; with high temperatures reducing the antibacterial and wound-healing properties of the honey. This is not surprising, as one of the main components of honey is Sucrose, which is composed of Carbon, Hydrogen and Oxygen. Upon heating, starting at 85 °C, the sugar breaks down to form carbon dioxide and water. At 110 °C the sucrose breaks down to glucose and fructose and as water is lost oxidation and degradation occurs^40,41^. By the hard crack (HC) stage, the solution is supersaturated and solidifies at room temperature. During the heating process, a Maillard reaction occurs due to the acidic pH (~ 4), which results in browning. Heating can also result in the formation of hydroxymethylfurfural (HMF), which has been shown to have potential cytotoxic and mutagenic properties at high concentrations^41–43^.

It is not surprising that different concentrations of honey were necessary to achieve the optimal efficacy of wound healing and bacterial killing. The concentrations identified in this set of experiments is consistent with the previous literature^44–46^. However, one should not assume that the in vitro concentration directly translates to the in vivo application of the MHMs. There are many things to take into account, including the rate of honey absorption into the surrounding tissues as well as the interaction with the immune system and local environment. One example of this is the 100% pure Manuka honey applied to various wounds, demonstrating enhanced wound healing, no cytotoxicity and progression of the lesion. In the continued development and optimization of MHMs, there may in fact be many formulations that are developed and which are optimal for different use cases, such as a high concentration honey for a contaminated or infected wound versus a low concentration honey for a clean wound^47–49^. It is also important to note that this preparation method is not limited to Manuka honey, as this low-temperature dehydration protocol would enable the production of microneedles from various types of honey. In addition to Manuka honey, other honeys have been shown to exhibit antimicrobial potential, including honeys from South Africa, Cameroon and Saudi Arabia (not an all-inclusive list), which are likely also have heat-sensitive antimicrobial activity^50–55^.

Additionally, with the optimization of the MHM conditions, low pressure and low temperature, other scaffolding materials or additives can be included in the formulation, allowing honey to be a new sugar-base for microneedle synthesis^56,57^. Future versions of the MHMs can be customized and include other substances or drugs, such as specific antibiotics or aloe vera, for synergistic effects^58,59^. For example, Manuka honey has been shown to act synergistically with phage therapy^60,61^. MHM-drug combinations may serve as new treatment modalities for conditions such as granulomatous TB, where the honey can be combined with phage therapy and be delivered directly into the site of infection/granuloma. This same approach could be used for cancer therapeutics and local administration of honey and other drugs to solid tumors^62–64^. The immunomodulatory properties of the honey could also stimulate a “cold” tumor to switch to “hot”. In fact, Manuka honey has been shown to stabilize proteins, which may put MHMs in a unique position to serve as a good drug delivery substrate, or even vaccine delivery—both preserving the protein to be delivered, while stimulating a local immune reaction or even serving as a natural adjuvant, promoting rapid healing of the microneedle injection site and help preventing bacterial infection of the injection site^65,66^. Such a delivery mechanism can also open the door to new ways of mucosal delivery of therapeutics or vaccines.

## Study limitations

In this study of the development of MHMs, although many interesting questions have been answered, many follow. It will be essential to follow-up with a thorough evaluation of the chemical composition and enzymatic activity of the honey after it is subject to the various preparation conditions, as this will enable a more in-depth understanding of the behavior of the MHMs in vitro and in vivo. In this study, only the bactericidal effects of the honey over 24 h were evaluated. In future studies, this bacterial-honey interaction should be further parsed, and the bactericidal versus bacteriostatic effects should be studied and the MIC of the MHMs should be determined^67^. Similarly, the wound-healing experiments were only of dermal fibroblasts, and other cell types should be studied as well as the migration along with the presence of an infection. Additionally, one of the most important property of microneedles is tensile strength and dissolution kinetics when punctured through the skin (or any other tissue it is applied to). Future studies should characterize the physical properties of these MHMs and in vivo testing should be performed to assess the effect of the combination of micro-needling and Manuka honey.

It is important to note that honey is a complex solution with hundreds to thousands of chemicals and compounds that interact with the local tissue and cells in a variety of ways. The honey likely interacts with the local cells, stimulates them through various signaling mechanisms, and then these cells further signal to other local and migrating cells. For example, when honey is applied to a wound bed, such as an ulcer or non-healing wound, some of its’ observed functions include: autolytic debridement, wound healing, inflammatory modulation and bacterial killing^12,68,69^. How all of these functions are achieved with the application of high concentrations of honey, > 50%, when it is observed in vitro that > 1% of honey is cytotoxic, supports the idea that we do not fully understand all of the mechanisms and chemicals within this “neutraceutical”. We suspect that how the honey interacts with the local cells will also be highly dependent on the rate of diffusion of the honey into the tissue from the MHMs, as natural clearance of the honey will also expose the tissue to a concentration gradient over time, resulting in various simultaneous physiological effects. Additionally, the active chemical compounds present within the honey likely also dictate the functional effects of application onto various biological environment^70^. We must also take into consideration the combinatorial, and potentially synergistic, effects on cell signaling and response when stimulated by both the honey and the physical act of microneedling, which has been shown to promote wound healing and reduce scar formation. 

### Conclusions

This study was done in order to optimize Manuka honey microneedle synthesis, while maintaining the Manuka honey’s natural antibacterial and wound-healing properties. Through a series of calculations and characterizations, we have developed a protocol for the conversion of Manuka honey from a thixitropic liquid to a supersaturated sugar solution with < 1% moisture content; allowing for the molding of the honey into hard crack microneedles. By using a low pressure and temperature approach, we also demonstrate the retained biologic activity of the honey and the detrimental effect of high heat on the honey. Based on these results, the next set of experiments should include, both, physical characterization of the microneedles, as well as testing in in vivo models. Using this new honey-based microneedle synthesis approach, honey can now be used as a primary or adjunct scaffolding component within microneedles, and can even be combined with other substances for additive effects. Using this new honey-delivery approach, Manuka honey may now be studied in the context of the treatment of bacterial infections on or below the skin barrier.
